# Text analysis of rehabilitation nursing policy in China from the perspective of policy tools

**DOI:** 10.3389/fpubh.2025.1562889

**Published:** 2025-05-01

**Authors:** Wenjia Li, Yuhan Zhu, Xuehua Zhu

**Affiliations:** School of Nursing, Zhejiang Chinese Medical University, Hangzhou, China

**Keywords:** policy tools, rehabilitation care, nursing policy, text analysis, public health

## Abstract

**Objective:**

This study aims to analyze the specific content and structural characteristics of rehabilitation nursing-related policies in China from 2007 to 2024, utilizing the perspective of policy tools. The goal is to provide insights for the subsequent optimization and enhancement of the rehabilitation nursing policy system.

**Methods:**

According to the classification method of policy instruments proposed by public policy scholars Rothwell and Zegveld, this study identified the X dimension (basic policy instrument) and categorized the included policies into demand-oriented, supply-oriented, and environment-based policies. Simultaneously, in light of multi-level service needs, an analysis of the elements constituting the rehabilitation nursing system was conducted in conjunction with existing research. This led to the summarization of five levels within the rehabilitation nursing system: nursing staff, service objectives, service items, service methods, and safeguard measures. These levels were designated as the Y dimension (service system) for this study. Utilizing this two-dimensional analytical framework, researchers classified and coded relevant policy texts that met established criteria. Subsequently, they analyzed their content and performed a quantitative assessment regarding both quantity and distribution patterns.

**Results:**

A total of 41 policy documents were analyzed, resulting in the extraction of 292 policy terms, and in dimension X, environment-based, supply-based, and demand-based policy instruments comprised 46.58, 36.64, and 16.78%, respectively. In dimension Y, nursing staff, service objectives, service items, service methods, and safeguard measures accounted for 22.95, 12.67, 12.67, 20.89, and 25.00%, respectively. Overall, the rehabilitation nursing policy in China is predominantly characterized by environment-based policy tools, while demand-based policy tools are comparatively underrepresented.

**Conclusion:**

The government has increasingly prioritized rehabilitation nursing care; consequently, the current policy framework is evolving toward greater specialization, standardization, and systematization. Nevertheless, there remains a need to optimize the structural application of policy tools further, particularly concerning enhancing the utilization of demand-side policy instruments.

## 1 Introduction

With the global population aging at an accelerated pace, the demand for rehabilitation care is rapidly increasing across numerous countries (1). For example, the United States and Japan, as representatives of developed nations, have advanced their rehabilitation care services through initiatives such as the Older Americans Act and long-term care insurance systems (2). However, China faces more complex challenges in this domain. On one hand, its older adult population is significantly larger; on the other hand, disparities in economic development and healthcare resource distribution between urban and rural regions hinder both accessibility to and quality of rehabilitation care. As of 2023, China’s population aged 65 and above has reached 220 million (3), surpassing the combined older adult populations of the United States and Japan. This positions China among those countries with the highest global demand for rehabilitation care (4). This study examines the distribution and characteristics of policy instruments within China’s rehabilitation care framework, providing a case study on how an emerging economy addresses the challenges posed by an aging population while offering valuable insights for other developing nations.

Rehabilitation nursing is a comprehensive service tailored for individuals who are ill, injured, or disabled, emphasizing holistic, continuous, and diverse care (5). At the 2024 National People’s Congress, China reaffirmed the strategic significance of rehabilitation medicine. As an essential component of rehabilitation medicine, rehabilitation nursing not only concentrates on disease treatment but also aims to restore patients’ capacity for independent living (6). Its services cater to all age groups, with particular emphasis on the older adult population. Although rehabilitation care policies are increasingly critical in enhancing public health and addressing the challenges associated with aging, international research has predominantly concentrated on care practices, technological advancements, and patient outcomes (7, 8). Systematic analyses of policy structures and the distribution of policy tools remain limited, particularly those employing textual analysis through the lens of policy instruments. In contrast to the well-established and systematic policy frameworks found in developed countries, developing nations—struggling with transformations in their health service systems—have experienced relatively little comprehensive exploration of rehabilitation care policies (9, 10). This study seeks to fill this gap by analyzing Chinese rehabilitation care policy texts from 2007 to 2024. It identifies key characteristics, distributions of policy tools, and developmental trends, thereby offering new perspectives for international research while providing both theoretical and practical references for optimizing global health policies.

## 2 Materials and methods

### 2.1 Search strategy

The primary source of policy data for this study is the “Peking University Legal Information Network.” In alignment with the research topic, keywords such as “rehabilitation nursing” and “rehabilitation services” were utilized as titles, complemented by terms like “nursing”, “care”, “support”, and “assistance” as full-text keywords. To ensure comprehensive coverage of relevant policies, additional documents were sourced from official websites, including the Chinese Government website and the National Health Commission, particularly focusing on their policy and regulatory sections. This methodology was adopted to guarantee both the authority and timeliness of the included policy documents. In this study, rehabilitation care policies are defined as those issued at the central government level that directly or indirectly facilitate the advancement of rehabilitation care services. The search was conducted up to 2024.

### 2.2 Inclusion and exclusion criteria

Inclusion criteria:

(1) The policy must be issued by government entities, such as the National Health Commission. (2) The content of the policy should be closely related to rehabilitation nursing. (3) The document must be a formal government publication, which includes laws, regulations, plans, opinions, notices, etc. (4) The policy should be current and reflect the latest developments in China’s rehabilitation care sector.

Exclusion criteria:

(1) Policies categorized as “responses, ““replies, “or “letters” are excluded. (2) Documents with lower authority status, such as advisory documents, bulletins, or briefings will not be included. (3) Any documents that merely mention keywords without providing substantial content are excluded. (4) Duplicate documents will also be disregarded.

Based on these criteria, a total of 41 policy documents were ultimately included; some of these are listed in Table 1.

**Table 1:** Policy documents related to rehabilitation nursing (part).

No.	Policy name	Legal authority level	Issuing institution	Publication date
1	Nurse Training Guidelines in Specialty Care	Departmental Regulatory Document	Ministry of Health (Disbanded)	May 25, 2007
6	“12th Five-Year Plan” for the Development of Disabled People’s Affairs	State Council Regulatory Document	State Council	May 16, 2011
		…		
19	Establishing and Improving the Elderly Health Service System	Departmental Regulatory Document	National Health Commission; National Development and Reform Commission, and 8 other ministries	October 28, 2019
31	National Nursing Career Development Plan (2021–2032)	Departmental Work Document	National Health Commission	April 29, 2022
33	“14th Five-Year Plan” for Healthy Aging	Departmental Work Document	National Health Commission; Ministry of Education; Ministry of Science and Technology, and 15 other ministries	February 7, 2022

### 2.3 Policy analysis framework construction

Different policy sub-goals necessitate distinct types of policy instruments, each yielding varying effects on these sub-goals. This study adopts the model proposed by public policy scholars Rothwell and Zegveld, which categorizes policy instruments into three primary types: supply-type, demand-type, and environment-type, with each type further subdivided into specific subtypes (11). To address the necessity for tailored services that cater to diverse groups, conditions, and rehabilitation needs within a multi-level service demand context, this study employs literature analysis (12–14) to classify the rehabilitation care system into five dimensions: caregivers, service goals, service programs, service methods, and safeguards. These dimensions constitute the analytical framework for the Y-axis and provide insights into the developmental trajectory of rehabilitation care policies while addressing critical challenges in this field.

**Figure 1:**
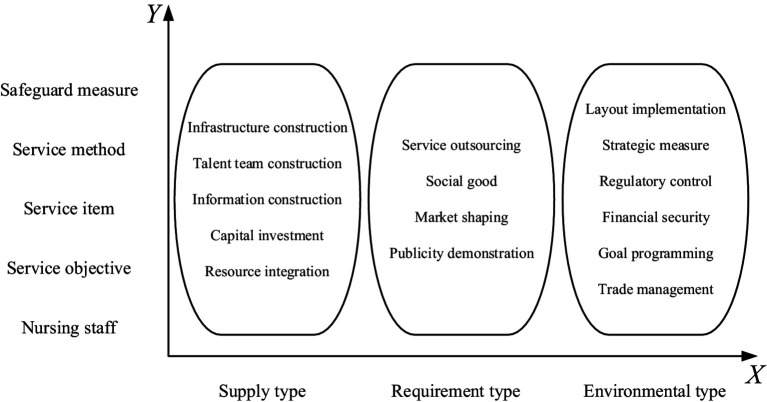
Two-dimensional analytical framework for rehabilitation care policy.

Building upon this foundation, a two-dimensional policy analysis framework has been developed. This framework comprises an *X*-axis (policy instrument dimension) and a *Y*-axis (service demand dimension), as illustrated in Figure 1. It offers a systematic approach to understanding and evaluating the role of policy instruments in achieving specific sub-goals; thus serving as a valuable tool for guiding both policy formulation and implementation.

### 2.4 Literature screening and information extraction

In this study, the policy text analysis method was employed, utilizing NVIVO 12.0 for information extraction and quantitative analysis of the selected policy texts. The two researchers independently extracted information from the screened policy documents, focusing on articles related to rehabilitation nursing policies as their content analysis unit. Policies were systematically numbered in a “Policy number - Chapter - Clause serial number” format, and the researchers categorized them according to dimensions of the policy tool and service system, resulting in a total of 292 codes. Prior to commencing the study, researchers underwent training to ensure consistency and stability in the results obtained. In instances where discrepancies arose during the coding process between the two researchers, a third researcher was consulted following discussion to achieve consensus. The coding process included cross-checking for consistency and reliability to guarantee both accuracy and validity in the analysis. Examples of text content coding pertaining to rehabilitation nursing policies are presented in Table 2.

**Table 2:** Examples of text coding of rehabilitation nursing policies.

Policy ID	Policy title	Policy content	Policy code	Tool type	Tool name	Service system
31	National Nursing Development Plan (2021–2034)”	Conducts job-specific training for nurses in critical shortage specialties, including pediatric care, intensive care, infectious disease care, rehabilitation care, and emergency care.	31-4-2-4	Supply-type	Workforce Development	Nursing Workforce

## 3 Policy coding analysis results

### 3.1 General information on policies

This study analyzed policy documents related to rehabilitation care published between 2007 and 2024. (1) In terms of the volume of issued documents, the highest number was recorded in 2022, with a total of six publications. This increase may be attributed to the ‘14th Five-Year Plan for National Health’ released by the General Office of the State Council in 2022, as well as the ‘14th Five-Year Plan for the Development of Health Care Personnel’ issued by the National Health Commission. The publication of these plans outlines a strategic direction for the development of the rehabilitation nursing sector, aiming to enhance the quality of rehabilitation nursing services and provide guidance and support to better address societal needs for rehabilitation nursing. (2) From an overall perspective, the number of policies related to rehabilitation care has shown a consistent upward trend. This indicates that the government is increasingly concerned about the development of the rehabilitation care industry and is actively formulating and implementing relevant policies to provide robust support for its growth. The sustained attention and backing from these policies have attracted more talent and funding into the field of rehabilitation nursing, thereby facilitating further development and expansion within the industry. (3) In terms of issuing bodies, most policies are primarily released by authoritative organizations such as the General Office of the State Council and the National Health Commission. Additionally, there are instances of joint issuance among multiple departments; for example, 15 ministries and commissions—including the National Health Commission, Ministry of Education, and Ministry of Science and Technology—collaboratively issued the “14th Five-Year Plan for Healthy Aging Notice.” Such interdepartmental collaboration fosters resource integration and synergy across various sectors, promoting the establishment of a mature and systematic policy framework that contributes to both national development and enhancement in the rehabilitation nursing care industry.

### 3.2 X-dimension analysis results

Statistical analysis revealed significant differences in the utilization of relevant policy tools within the realm of rehabilitation care. Specifically, supply-type, demand-type, and environment-type policy tools accounted for 36.64% (107/292), 16.10% (49/292), and 46.58% (136/292) respectively. Among these, environment-based tools were employed most frequently; within this category, target planning constituted the largest proportion at 13.36%, while financial security represented the smallest share at 4.11%. Supply-based tools ranked second in usage frequency, with human resource development being the most utilized tool at 15.41%, whereas financial investment accounted for only 1.03%. Demand-based tools were used least often; market shaping was the predominant tool in this category at 7.88%, while social welfare comprised merely 1.71%. These findings are summarized in Table 3.

**Table 3:** Distribution of policy instruments (*n* = 292).

Policy tool type	Policy tool name	Frequency	Proportion	Total count
Supply type	Infrastructure Development	16	5.48%	109 36.64%
	Talent Building	45	15.41%	
	Information construction	11	3.77%	
	Capital Investment	3	1.03%	
	Resource Integration	34	10.96%	
Demand type	Service Outsourcing	6	2.05%	47 16.10%
	Social Welfare	5	1.71%	
	Market Shaping	23	7.88%	
	Publicity and Demonstration	13	4.45%	
Environment type	Layout and Implementation	18	6.16%	136 46.58%
	Strategic Measures	19	6.51%	
	Regulatory Control	16	5.48%	
	Financial Security	12	4.11%	
	Target Planning	39	13.36%	
	Industry Management	32	10.96%	
Total	–	–	–	292

### 3.3 Results of Y-dimension analysis

According to the statistics regarding the number of policy tools within the service system dimension, as presented in Table 4, it is observed that 22.95% of these policy tools pertain to nursing staff, 12.67% relate to service objectives, 20.89% are associated with service items, 25.00% concern service methods, and 18.49% involve safeguards. The distribution of each element appears generally balanced; however, there is a relatively small proportion of policy tools related to the objective elements of rehabilitation care services, indicating a need for further clarification and specification in this area.

**Table 4:** Distribution of items in dimension of service needs (*n* = 292).

Service requirements	Specific elements	Frequency	Percentage
Nursing staff	The requirements for the demand, allocation, training and professional competence improvement of rehabilitation nursing staff in the policy. To explore how to cultivate, introduce and retain professional nursing staff to ensure the quality and coverage of rehabilitation nursing services.	67	22.95%
Service objectives	To improve the quality of life of those in need of rehabilitation, reduce family burden, and promote the functional recovery of patients. To explore how to set the goals of rehabilitation care for various groups and ensure the realization of these goals.	37	12.67%
Service items	Basic rehabilitation nursing service, rehabilitation nursing training and knowledge service, mental health care service and other nursing services for the purpose of promoting the rehabilitation of the service object.	61	20.89%
Service methods	The innovation and application of the policy in service methods, including personalized nursing, interprofessional cooperation, intelligent health management and other service methods, how to meet the needs of patients at different levels and improve the effect and efficiency of rehabilitation nursing.	73	25.00%
Safeguard measures	The measures taken in the policy to ensure the implementation of rehabilitation nursing services, including financial support, legal protection, social support, supervision mechanism, etc., improve the sustainability and effect of the service.	54	18.49%

### 3.4 Results of X-Y dimension analysis

A comprehensive analysis of the X-dimension of basic policy tools and the Y-dimension of service systems reveals, as illustrated in Table 5, that supply-type policy tools place greater emphasis on the element of caregivers, with a frequency count of 47. In contrast, environment-type policy tools focus more on the elements related to service items and service methods, recording frequencies of 34. Demand-type policy tools prioritize service methods and safeguards, with frequencies of 16 and 13, respectively.

**Table 5:** L-shaped matrix of policy tools and service system elements (*n* = 292).

Instrument of policy	Demand for services				
	Nursing staff	Service objectives	Service items	Service methods	Safeguards
**Supply type**	**47**	**9**	**19**	**23**	**11**
Construction of infrastructure	0	0	7	3	6
Talent team building	45	0	0	0	0
Information building	0	0	3	8	0
Capital input	1	1	0	0	1
Resource integration	1	8	9	12	4
**Environmental type**	**17**	**21**	**34**	**34**	**30**
Layout implementation	0	4	4	8	2
Strategic measures	2	4	4	5	4
Regulatory control	5	2	1	0	8
Financial protection	1	2	2	2	5
Goal planning	2	9	14	10	4
Industry management	7	0	9	9	7
**Demand type**	**3**	**7**	**8**	**16**	**13**
Outsourcing of services	0	1	1	2	2
Social good	0	1	1	0	3
Market shaping	1	2	4	12	4
Promotional demonstration	2	3	2	2	4

The bold values represent the frequency of three different policy tools in the policy.

The varying emphasis on different elements within service systems across distinct types of policy tools suggests that when formulating policies, the government carefully considers the unique characteristics associated with each type to select the most suitable tool for specific policy objectives. This differentiation in focus among various types of policy instruments underscores how governmental decision-making is informed by an understanding of these characteristics, ultimately guiding them toward choosing the most appropriate instrument for achieving targeted outcomes.

## 4 Study conclusions and recommendations

### 4.1 Differences in the use of various types of policy instruments and structural optimization

This study revealed significant disparities in the utilization of three policy tools—environmental, supply, and demand—in rehabilitation nursing policies. Notably, environmental policy instruments accounted for the largest share (46.58%), indicating that the government places considerable emphasis on fostering a conducive development environment for the rehabilitation nursing industry. A favorable industry environment not only enhances the motivation of nursing staff but also ensures high-quality rehabilitation nursing services (15, 16). Conversely, while there was a relatively high frequency of use for supply-oriented policy tools (36.64%), applications related to infrastructure, information dissemination, and financial investment remained inadequate—particularly within community nursing services. The lack of personalized service offerings represents an urgent issue that requires resolution (17). Demand-oriented policy tools were utilized the least frequently (16.10%), which may be attributed to the developmental stage of China’s rehabilitation care industry. In comparison to the United States, rehabilitation nursing in China commenced later and has progressed slowly; moreover, the concept of rehabilitation nursing remains largely unrecognized among the general population. This is particularly evident in the insufficient attention given to the complex needs of middle-aged and older adult individuals, as well as patients with chronic diseases (18, 19). Despite having a considerable number of rehabilitation institutions, China faces several challenges including low service rates, disorganized operational models, inadequate facilities, and insufficient personnel training (20, 21). The existing social conditions interact negatively with the infrequent application of demand-oriented policy tools, resulting in an imperfect allocation of rehabilitation resources. Consequently, some patients are discharged directly after their acute phase without receiving systematic rehabilitation treatment at home, which significantly increases their risk for postoperative complications (22).

Therefore, it is crucial to draw upon existing achievements and experiences to optimize medical resource allocation and alleviate pressure on large hospitals. Implementing a three-tiered medical referral mechanism could effectively direct patients with varying conditions to appropriate specialized institutions for tailored treatment and care (23, 24). Furthermore, there is a pressing need to enhance standardized management practices, guide quality service outsourcing initiatives, promote high-quality policy tools, and rectify prevailing industry disarray. In light of the multi-level demand context, the government should optimize the structural utilization of various policy tools and reasonably allocate the proportions of environmental, supply-based, and demand-based instruments. In applying environmental policy tools, it is essential for the government to focus on establishing a conducive policy environment and a robust industry development framework. This includes providing enhanced support in terms of capital investment, legal regulations, and industry management (25). In relation to supply-based policy tools, there should be an emphasis on cultivating rehabilitation nursing professionals and developing infrastructure to adequately meet the diverse needs of patients at different levels (26). Regarding demand-based policy tools, the government ought to strengthen guidance for social capital involvement as well as support for social welfare initiatives. Furthermore, it is crucial to encourage multiple societal forces to engage actively in the construction of rehabilitation nursing services (27).

### 4.2 Distribution and optimization suggestions of service demand elements

This study introduced a Y dimension based on the X dimension to construct a two-dimensional framework for analyzing rehabilitation nursing policy texts. In the service needs dimension, the research findings indicated that elements related to service methods accounted for the highest proportion, reaching 25.00%. This result underscores the government’s significant emphasis on developing and implementing effective rehabilitation nursing service methods, highlighting the importance of providing support through these methods to achieve improved rehabilitation outcomes and service quality. Additionally, nursing staff and service items represented 22.95 and 20.89%, respectively, which aligns with existing research findings. The study demonstrated that training nursing staff in professional skills and competencies is a crucial prerequisite for enhancing the quality of care provided in rehabilitation settings (28). Conversely, elements pertaining to service targets constituted the lowest proportion at only 12.67%. Clearly defined service objectives are essential for delineating the scope of implementation in rehabilitation nursing and addressing the needs of those receiving services. Therefore, it is particularly necessary (29) to balance various factors according to actual circumstances, continuously enhance facilities within rehabilitation nursing institutions at all levels, and improve both education and professional standards among practitioners in order to establish a high-quality rehabilitation nursing team. Excellent talents are fundamental to the continuous progress of the industry. It is essential to reform the talent training system, focusing on international development trends while considering the actual circumstances. This involves actively exploring the construction of a new curriculum system and professional standards that align with the talent training objectives of medical colleges and universities (30). Furthermore, it is crucial to establish and enhance professional training programs that better meet the industry’s actual needs and closely correspond with the requirements of international rehabilitation industry support courses.

It is widely recognized that different groups have varying needs for rehabilitation care. To address these diverse requirements across different demographics and levels, it is recommended that policymakers strengthen service objective settings to ensure comprehensive coverage of needs at all levels (31). In light of multi-level needs, service goals should be articulated more clearly, with a focus on refining the rehabilitation nursing requirements for patients at different levels—ranging from basic health maintenance to personalized in-depth care (32). Policymakers should facilitate the coordination and integration of nursing staff training, service project design, and service objectives to ensure that policies effectively meet varying demands while simultaneously improving the overall efficacy and quality (33) of rehabilitation nursing.

### 4.3 Professional and systematic policy construction driven by multi-level service demand

Rehabilitation nursing, as a vital domain related to public health, is encountering an increasing array of multi-level needs. Currently, China has yet to establish a mature rehabilitation nursing industry system. The Outline of the Plan for the National Medical and Health Service System (2015–2020), issued by the General Office of the State Council in 2015, proposed promoting “the formation of a continuous service model encompassing diagnosis, treatment, rehabilitation, and long-term care.” This initiative aims to achieve a rational medical treatment framework characterized by “minor diseases managed at primary care facilities, serious illnesses treated in hospitals, and rehabilitation facilitated through primary care.” The document underscores the transitional role of rehabilitation nursing between hospital settings and home environments; particularly during patients’ recovery processes where home care can offer more personalized support (34). However, existing policies still exhibit a lack of clarity regarding both the temporal scope and service content associated with rehabilitation nursing. In light of diverse service demands across multiple levels, there is an urgent need for a more comprehensive collaboration mechanism.

The multi-level service demand indicates that rehabilitation nursing must not only address the fundamental rehabilitation needs of the older adult and disabled individuals but also extend to encompass chronic diseases, cancer, and other populations (35). This highlights the differentiated requirements for rehabilitation nursing across various groups (36). Therefore, it is recommended that the government enhance coordination among hospitals, communities, and families to promote a comprehensive and continuous service model (37, 38). This approach should particularly focus on achieving rational resource allocation and precise matching of needs at different stages of rehabilitation. Furthermore, the government should encourage the establishment of inter-agency collaboration mechanisms to facilitate effective information sharing and communication. Such measures will optimize service quality and improve resource utilization efficiency, thereby better addressing multi-level rehabilitation needs while promoting patients’ recovery and social integration (39).

Current policies primarily focus on the older adult and disabled populations, while insufficient attention is given to other groups with rehabilitation care needs, including individuals with chronic illnesses. Therefore, it is imperative for the government to clearly define the target population for rehabilitation nursing services, establish a robust service evaluation mechanism, and ensure accurate identification and assessment of service recipients. As high-quality nursing staff are essential in delivering effective rehabilitation nursing services, it is crucial to enhance the treatment of practitioners in order to attract more talented professionals into the field. It is recommended that welfare and security measures for these practitioners be improved. Concurrently, the government should bolster economic incentives within the industry through innovative mechanisms, resource integration, and diversified investment strategies (40). Such efforts will stimulate the engagement of stakeholders in social capital and effectively address multi-layered needs while ensuring that basic service requirements are met, thereby contributing to sustainable development within the sector. Adequate policy support plays a crucial role in the industry’s advancement. In 2021, China designated 15 provinces as pilot areas for rehabilitation medical services, with the government taking a proactive stance to promote the growth of the rehabilitation nursing industry (41). The study indicates that among the five provinces participating in this pilot initiative—Shandong, Jiangsu, Hunan, Guangdong, and Zhejiang—the distribution of rehabilitation institutions ranks among the highest in the country (42). However, it is insufficient to focus solely on developing rehabilitation nursing services in populous cities with high economic development; greater attention must be directed toward remote areas that lack adequate rehabilitation resources moving forward. Governments can implement capacity-building projects for less developed regions by adopting differentiated financial support policies. Furthermore, multi-sectoral coordination platforms can be established through integrated arrangements to ensure effective policy implementation across administrative boundaries.

## 5 Conclusion

Through a quantitative analysis of Chinese rehabilitation nursing policy texts, this study elucidates the distribution characteristics and potential issues associated with policy objectives and tools. The findings indicate that while China’s rehabilitation nursing policy system has seen gradual improvements in recent years, there remains significant room for enhancement regarding the clarity of objectives and the balance among policy instruments. In comparison to international rehabilitation nursing policies, China’s framework still requires strengthening in terms of service system improvement and diversification of tool utilization. This research not only addresses a gap in the quantitative analysis of domestic rehabilitation nursing policies but also offers a novel perspective for international policy studies. Moving forward, it is essential to further optimize policy design, enhance resource integration and service equity, thereby promoting the sustainable and healthy development of the rehabilitation nursing sector.

## Data Availability

Publicly available datasets were analyzed in this study. This data can be found here: https://www.gov.cn/.
